# Disaster Management System Aided by Named Data Network of Things: Architecture, Design, and Analysis

**DOI:** 10.3390/s18082431

**Published:** 2018-07-26

**Authors:** Abdul Hannan, Sobia Arshad, Muhammad Awais Azam, Jonathan Loo, Syed Hassan Ahmed, Muhammad Faran Majeed, Sayed Chhattan Shah

**Affiliations:** 1 Department of Computer Engineering, Faculty of Telecom and Information Engineering, University of Engineering and Technology (UET), Taxila, Punjab 47080, Pakistan; hannan.comsats@gmail.com (A.H.); awais.azam@uettaxila.edu.pk (M.A.A.); 2 School of Computing and Engineering, University of West London, London W5 5RF, UK; jonathan.loo@uwl.ac.uk; 3 Department of Computer Science, Georgia Southern University, Statesboro, GA 30458, USA; sh.ahmed@ieee.org; 4 Department of Computer Science, Shaheed Benazir Bhutto University, Sheringal, Upper Dir 18000, Pakistan; m.faran.majeed@ieee.org; 5 Department of Information Communication Engineering, Hankuk University of Foreign Studies, Seoul 02450, Korea; shah@hufs.ac.kr

**Keywords:** NDN, IoT, Beacon Alert Message, Disaster Management System, Push-Support

## Abstract

Disasters are the uncertain calamities which within no time can change the situation quite drastically. They not only affect the system’s infrastructure but can also put an adverse effect on human life. A large chunk of the IP-based Internet of Things (IoT) schemes tackle disasters such as fire, earthquake, and flood. Moreover, recently proposed Named Data Networking (NDN) architecture exhibited promising results for IoT as compare to IP-based approaches. Therefore to tackle disaster management system (DMS), it is needed to explore it through NDN architecture and this is the main motivation behind this work. In this research, a NDN based IoT-DMS (fire disaster) architecture is proposed, named as NDN-DISCA. In NDN-DISCA, NDN producer pushes emergency content towards nearby consumers. To provide push support, Beacon Alert Message (BAM) is created using fixed sequence number. NDN-DISCA is simulated in ndnSIM considering the disaster scenario of IoT-based smart campus (SC). From results, it is found that NDN-DISCA exhibits minimal delay and improved throughput when compared to the legacy NDN and existing PUSH schemes.

## 1. Introduction

The Internet of Things (IoT) aims to provide promising connectivity solutions across the globe. IoT also plays a vital role for communication among the homogeneous and the heterogeneous devices required to control and monitor these devices. The main reason behind the attraction of researchers in IoT is smart devices that are frequently used nowadays [1,2]. By enabling IoT, we can build smart applications such as smart health-care [3], smart grid, smart home [4,5], smart campus [6,7,8], smart building [9] and smart city [10] with more reliability. One of the major applications of IoT is Disaster Management System (DMS). DMS mainly considers flood, earthquake, fire, and storm. Many organizations are working to build such IoT-based DMSs that include ZIZMOS funded by SBIR [11] and RIO operation center funded by IBM [12]. Disaster (fire) management becomes more effective for the crowded places such as smart campuses, shopping malls and airports. From IoT perspective, smart campus (SC) is an important scenario. In SC, thousands of students move around the campus premises during a day. It becomes significant as well as difficult to protect each and every student from such a disaster. For example, a disaster (fire) incident occurred due to a short circuit in a classroom of The University of Lahore (UOL) Islamabad campus and another tragic fire disaster occurred at St. Andrew University United Kingdom in its chemistry department [13]. In few seconds, the whole department was on fire. Moreover, the information loss along with infrastructure damage were added effects [14] of that disaster. However, it could be beneficial if university administration installs an IoT-based DMS on the campus to protect students’ life. In order to make an IoT-based DMS for an SC, we need a robust and reliable communication among IoT devices. List of key terms which we use in this paper are listed in Table 1.

The existing IP architecture is less suitable for IoT-based DMS due to myriad reasons [15] that include lack of the fool-proof security (but as an additional patch in the form of IP-Sec that increases overhead), lack of the addressing mechanisms for the IoT devices and naming of humongous information produced at every second, no built-in support for mobile devices (but in the form of patch as mobile-IP), no mechanism of network fragments, exhaustive memory and power consumption due to heavy TCP/IP on small devices and bandwidth utilization, and large sizes of IP packets due to inclusion of address of the corresponding device (which is not needed when the user is only interested in data) [16,17,18,19].

On the other hand, Information-Centric Networking (ICN) architectures are being proposed in the last two decades. There are eight ICN-based architectures proposed so far for static and mobile networks such as DONA, NetInf, CCN, NDN, MobilityFirst, PURSUIT, CONVERGENCE, and COMET [20,21]. ICN architectures deal with the name-based contents as the main component in communication instead of IP address. With ICN naming of smart devices in the IoT applications, ICN naming plays a significant role by considering addresses of both the IoT sensors and the actuators as named-content along with the data they provide as named content. It is quite useful to manage IoT infrastructure as content-centric than host-centric TCP/IP architecture [16,18]. ICN-based architectures (more specifically Name Data Networking (NDN)) have interest and data messages that smart (networked) devices can use to inquire and get a response from the IoT network [22]. To provide absolute security, both of these messages are signed by the consumer and the provider of the information. Moreover, issuance of any information is only initiated whenever the consumer requests for the data which makes it further secure. Furthermore, re-registration of device’s corresponding information, with the new nearby connecting devices, handles mobile devices more easily. ICN models offer scalable and cost-effective content distribution due to caching and name-based networking [22].

Based on the above-mentioned advantages of ICN for IoT, we consider the named data networking (NDN) as an ideal choice to build IoT-based DMS architecture. Moreover, in NDN by default the PULL support scheme works between consumer and producer. In PULL support, the consumer sends the interest message (IM) and in response producer replies with a data message (DM) for the requested interest [20]. PULL support is not feasible and fruitful in an alarming situations and disasters such as fire, flood and earthquake. In such situations, we need robust and efficient communication with the minimal delay. We can not afford to wait for a particular interest from the consumer to get the response from the producer. There is another communication scheme known as PUSH support came into existence that tackles the alarming and disastrous situations. In PUSH support, interest is forwarded through producer without any consumer on the basis of the disaster monitoring sensors [23]. To discuss the significance of push-based NDN, various approaches are implemented in the scenarios of Vehicular Ad hoc Networks (VNDNs), IoT and many more. It is worth noticing that our NDN based DMS scheme, is specifically related to the scenario that contains disastrous incidents. In such incidents, the long-lived-interest mechanism is infeasible as keeping such interests will bring extra overhead and such interests would be replaced as long as the timer of those interests expires. Handling unsolicited data or content is previously done by Majeed et al. [24], in which the authors have devised a scheme of pre-caching the content by generating temporary interests for handling unsolicited data so that it is not discarded on arrival by the content router or the consumer. This scheme is especially designed for multimedia data and authors have not considered the critical nature of the content. In such incidents, the major drawback of unsolicited data and long-live-interest push schemes is retransmission of packets at regular intervals. It is due to the request time out (RTO) and virtual request time out (vRTO) factors. It also add additional overhead in the payload field in the data and interest packets [23]. However in DMS, the overhead of these computations increases the overall delay of packets to propagate from one node to another node. It also decreases the overall throughput in a SC which is not affordable in real life scenarios. Furthermore, in IoT-based DMS scenario which has smart gadgets with limited memory and power, it is not feasible to perform push transmission through these existing schemes. For NDN-IoT based DMS, we need an efficient system which utilize less memory and attain higher throughput. Thus in our NDN-IoT based DMS scheme, we propose a lighter version of PUSH support to incorporate above-mentioned issues (of throughput and delay) through generation of synthetic interest based BAM. In our scheme, we don’t need re-transmission of packets due to time out factor as in previous PUSH schemes [23]. Secondly, we consider only one fixed sequence number (seq. no) in a disaster mode, so it minimizes the overall BAM (transmission and propagation) delay of a DMS. Thirdly, it also increases throughput of DMS by less number of discarded bits. Finally, none of the existing PUSH schemes are proposed for the disaster management application via NDN, so our propose scheme is unique in itself.

**Figure 1:**
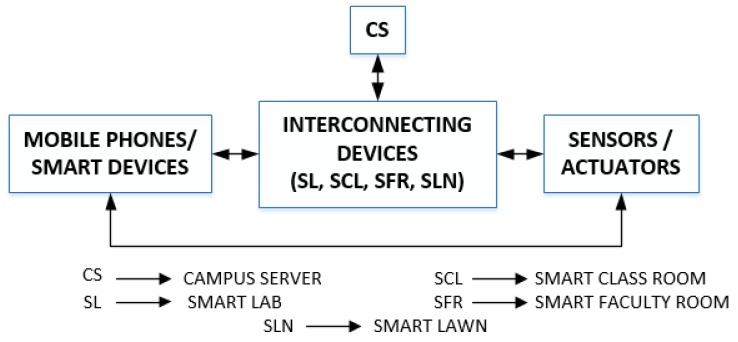
Proposed NDN Based Disaster Management System for Internet of Things based Smart Campus Architecture (NDN-DISCA).

By considering all loopholes in the existing work, we propose an architecture for IoT-based DMS for smart campus (SC) with the help of NDN architecture. We call our propose architecture as NDN-DISCA. It involves fire sensors, actuators, smart campus user (SCU) mobile phones, Wi-Fi interconnecting devices, and a campus server (CSR). Wireless fidelity interconnecting devices are further installed into five partitions; smart lab (SL-1, SL-2), smart faculty room (SFR), smart classroom (SCL) and smart lawn (SLN). We use fast dissemination, a naive characteristic of NDN [25], for the transmission of critical data to build NDN-DISCA. In NDN-DISCA, critical information travels from the fire sensors towards the interconnecting devices (Wi-Fi nodes) then SCU mobile phones and ultimately moves toward the CS. Our proposed scheme NDN-DISCA is shown in Figure 1.

We propose a lighter version of beacon-based PUSH support named as beacon alert message (BAM). It is used to detect fire disaster through NDN architecture in a IoT environment by considering dedicated fixed seq no. location i.e., “0” in our case. It requires less memory and dissemination power, hence it is appropriate for the IoT environment.

The major contributions of this paper are as follows:

1. We introduce the NDN architecture in an IoT based DMS and highlight its effectiveness in terms of scalability and content based communication infrastructure.
2. We propose a lighter version for PUSH support on the basis of beacon message (BAM) for DMS in an IoT scenario SC, which is more robust and effective than the schemes which are mentioned in [23,26].
3. We simulate IoT based SC with distinct nodes (i.e., both stationary and mobile) distribution in different clusters.
4. We evaluate and compare our proposed scheme NDN-DISCA against relevant schemes from [23] through analytical and quantitative analysis in terms of average delay (AD) and average throughput (ATH) and finds NDN-DISCA more feasible for IoT-DMS-SC.

This paper is structured as follows. In Section 1, we highlight the importance of NDN-based DMS in a SC. Section 2 refers to related work in ICN and IoT in the context of disaster management. Section 3 discuses the native NDN mechanism. In Section 4, we describes the proposed scheme NDN-DISCA along with the performance metrics. In Section 5, we discussed the simulation scenarios to evaluate and implement the NDN-DISCA. In Section 6, we discussed the experimental setup for different clusters distribution in SC. Performance analysis along with results discussed in Section 7. Finally in Section 8, we conclude with a conclusion and future work.

## 2. Related Research Efforts

In co-reliance to the aforementioned discussion, ICN approach is fully capable of implementing a well-structured DMS in the SC. In an SC, there is a hustle and bustle of students. In this situation, if a disaster (fire) occurs, it puts a drastic effect on student’s life and a campus infrastructure. In ICN architecture, every piece of the object has a unique name, which is directly incorporated by applications for the retrieval of data. The information produced from the sensors and the actuators, can be treated as content [7,27]. In DMS-SC, information is transferred from students that occupy smart gadgets (mobile nodes) such as smart phones, smart watches and smart brands or (static nodes) such as tower, routers, and servers, in the hop-to-hop fashion. Ultimately, it reaches the CS. Among the two most efficient ICN architectures named as NDN and CCN, for the mobile network, NDN architecture [19,20] is the best suitable architecture for the efficient implementation of DMS in a SC. It is due to minimum cache latency and get rid of interest looping problem as compared to the CCN architecture [27].

Meanwhile, Amadeo et al. [23] proposed three PUSH-based schemes named as interest notification, unsolicited data, and long lived interest. As discussed previously, in such incidents the long-lived-interest mechanism is in-feasible as keeping such interests will bring extra overhead and also such interests would be replaced as long as the timer of those interests expires. Handling unsolicited data or content is previously done by [24], in which the authors have devised a scheme of pre-caching the content by generating temporary interests for handling unsolicited data. So, it is not discarded on arrival by the content router or the consumer. This scheme is especially designed for multimedia data and authors have not considered the critical nature of the content.

In co-reliance, Majeed et al. [26] specifically target communication between nodes that become difficult, due to vigorous topologies of the vehicular ad-hoc network. The authors proposed a beacon messages-based PUSH support in a VNDN environment that utilizes less memory and is more flexible than the existing pull mechanism of VNDN. The major limitation of this paper is that it is specific to VNDN network not considering the NDN-IoT environment. Beside this, PUSH-based VNDN scheme also consumes more memory and power utilization while utilizing this PUSH support mechanism in the IoT environment. It is because author considers the data in the form of multimedia stream which includes additonal payload effect on IM. It is another drawback of this scheme.

However Seedorf et al. [28] enable communication in a delay tolerant network (DTN) [29,30] using ICN. This paper especially focused on PULL support-based disaster scenarios in which movement of the data mules are random and unpredictable [31]. In such scenarios, it is very useful to establish a mechanism through which one can prioritize or popularize the ICN interest messages [32] in a decentralized manner that is more reliable and flexible. Due to the complex memory utilization architecture in a disaster situation, this scheme is not preferable for IoT system. Moreover, nodes working in the IoT environment are heterogeneous with limited memory, minimal processing capacity, and limited power backup that cannot process the complex memory utilization algorithms in a real time. Due to this reason, the proposed scheme for a disaster situation is not preferable where robustness and effectiveness of the architecture are the major concerns for the rapid response. Parallel to this, Sourlas et al. [33] proposed PULL scheme-based information resilience scheme for CCN/NDN router architecture. The authors modified the existing NDN/CCN router architecture, with an amendment of satisfied interest table (SIT). SIT structure becomes functional when the network is temporary or partially fragmented. So, the user can retrieve cached content through SIT table. The authors also modified the interest packet structure for forwarding interests to the neighboring users when the network is fragmented or disruptive. We got greater throughput and information availability in a post disaster situation through SIT data structure. The major limitations of this paper, firstly it is only applicable in the non-IoT environment by considering only static nodes. Secondly, it is not implemented in a real-time for disaster detection. Disaster detection in the IoT architecture requires robust communication with a minimal delay in real-time. So, proposed SIT-based fragmented network scheme is not suitable in the IoT-based disaster detection process. In another work, Hasegawa et al. [34] proposed a PULL support NDN-based emergency message delivery mechanism centered on COPSS algorithm [35] with attribute-based message encryption facility [36]. In this paper, the authors not only proposed an alternative to the existing telephone system but also provided the resiliency to the failure [34]. The proposed mechanism is restricted to the NDN-based emergency message delivery scheme with the non-IoT environment. In order to detect disaster in IoT supported real-time environment, this scheme is not preferable because it is not capable to handle extensive IoT traffic in a disaster situation. In the IoT environment, every smart device communicates with another smart device for conveying information from source to destination in the hop-to-hop fashion. A large bundle of information is exchanged between the devices all the time in the IoT scenario. Without IoT infrastructure, it is not possible to deliver information with efficiency. In a disaster situation, we requires transmission of huge information from one device to another with minimal delay. The comprehensive comparison of our proposed scheme NDN-DISCA with the existing work also shown in Table 2.

## 3. Naive NDN Architecture

NDN is one of the most efficient, suitable and flexible ICN architecture, especially for IoT environment [37]. NDN model exhibits consumer producer paradigm which is more content centric than location-based IP model [18]. NDN communication is accomplished through two types of messages explicitly IM and DM [27], and three data structures namely content store (CS), pending interest table (PIT), and forwaring information base (FIB) for caching and forwarding the incoming IM [37,38]. Consumer broadcast the IM in the network to acquire entreated data and in response producer reply with produce data for the requested IM in the reverse path to the consumer node. IM contains the content name which identifies the requested data and DM contains both content name and content data along with the producer signature key, which is sent back to the consumer node. Intermediate routing nodes receive the incoming IM and forward the IM by looking up the names in the FIB data structure. Once the IM reaches the desired node, a DM is sent back to the consumer node.

**Figure 2:**
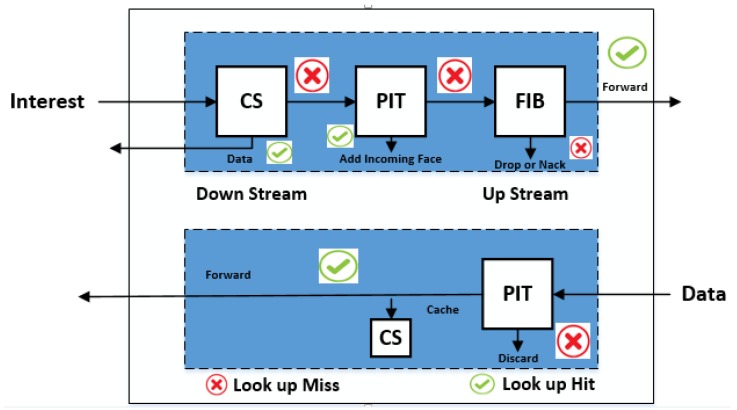
Two Way Packet Flow Communication in NDN.

In co-reliance to FIB, PIT data structure contains all the pending IM’s which are stored in queue, waiting for corresponding data. In case of multiple Interests for the same data are received from downstream, only a leading one is sent to the upstream towards data source. PIT data structure stores IM as a PIT entry which contains the content name of the interest along with the respective interest interface. Interface contains information regarding receivers from which interest for the identical IMs is recieved. When router receives corresponsing data, it finds the corresponding PIT entry and forwards the data toward all interfaces mentioned in its PIT entry. After this, router deletes the corresponding PIT entry in the PIT and caches the data in CS. As NDN communication model is independent of the content location, so data is cached in CS to entertain future interests [27] as can be visualized in Figure 2.

Moreover, naïve NDN supports PULL mechanism between consumer and producer through IM and DM [7,27,37,39]. PULL communication model requires an extensive delay in ACK and NACK validation process. Therefore, this NDN pull-based model is not feasible in the real world applications, where time critical information is one of the prime concern e.g., flood, earthquake and fire. As PUSH support mechanism is more robust and effective scheme, but it is less protected than PULL support model. In PUSH support model, producer broadcasts the unsolicited data based on the emergency situation, without any consumer IM request [23]. It is specially designed for situations, where forwarding of time critical information is the first the priority such as emergency alarms (flood, earthquake, and fire) which in return, require minimal delay and higher throughput [23].

While in IoT environment where multiple consumer nodes can be attached to a single producer node, we need a communication platform which support both PULL and PUSH mechanisms [4]. IoT in disaster situations needs a lighter version of NDN model with PUSH and PULL support enabled which should utilize less memory as compared to available NDN-based PUSH schemes like [4,23,26]. This is the main motivation and incentive behind our work. We propose an NDN-DISCA which is fully capable to effectively handle DMS than existing PUSH mechanism. In NDN-DISCA, we use fixed seq. no. for BAM to provide PUSH support. NDN-DISCA utilizes less memory as just one fixed seq. no. is consumed to save in it the PIT. Thus in these terms, it is a lighter version of PUSH support specially designed for IoT environment. Our proposed NDN-DISCA is more effective, scalable and flexible than existing schemes [4,23,26].

## 4. Proposed NDN Based DMS for IoT Based Smart Campus Use Case

In this Section, we discuss about the functionality of producer and consumer nodes in our propose scheme NDN-DISCA in both disaster and normal mode as discuss in Section 4.2 and Section 4.3. Moreover, we also discuss the BAM generation mechanism in NDN-DISCA in disaster mode refer to Section 4.3 along with the performance metrics such as AD, ATH and percentage throughput gain (%THG) to evaluate the proposed NDN-DISCA. Further, we also explain the selection of use-case adopted in our paper.

**Figure 3:**
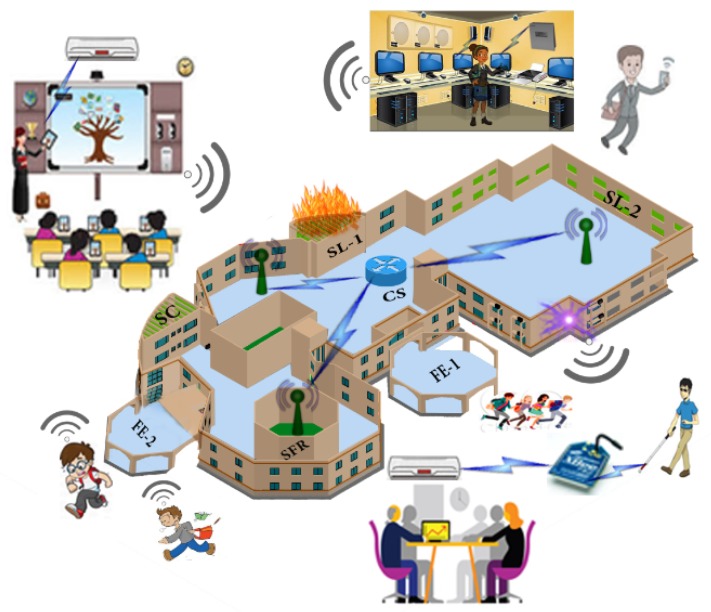
IoT Based SC.

The use-case consist of a SC that have different clusters such as SFR, SL-1, SL-2, SCL, Fire Exit (FE-1) and Fire Exit 2 (FE-2) is shown in Figure 3. It can be seen that in a situation when SC is under the fire disaster. Fire flames raising from SL-1 due to short circuit in electrical wiring. There is a main CSR which gathers information from multiple routers and Wi-Fi nodes available in its domain. Each router carries sensor information installed in its domain and forward it to the CSR. In normal mode, every sensor operates in under threshold limit (TL) of a fire disaster condition which disables disaster mode in smart devices. Whenever the sensor value goes beyond the TL, it indicates the existence of the fire situation anywhere under SC premises. In this situation, each smart device operates in disaster mode at which, transfer of critical information from one hop to another hop which ultimately reaches the CSR. This fire management methodology pre-alerts the nearby smart rooms. It alert students and teachers move towards a safe place before the fire disaster becomes uncontrollable and severe.

### 4.1. Overview of Proposed Scheme

**Figure 4:**
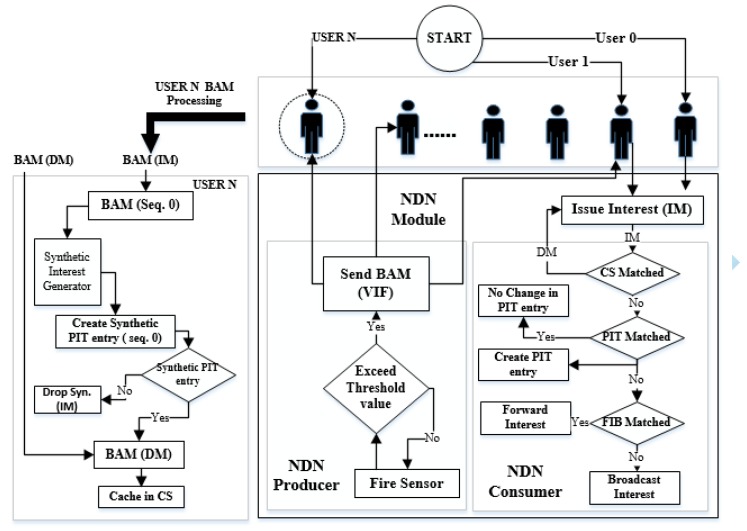
Proposed NDN-DISCA BAM(IM)- > BAM (Interest Message), BAM(DM)- > BAM (Data Message).

An NDN-IoT-based DMS in a SC (NDN-DISCA) is proposed in this paper, as shown in Figure 4. A specialize alert packet known as BAM is generated by a producer via Vanilla Interest Forwarding (VIF) broadcasting scheme on exceeding of TL of a fire sensor (FS) i.e., >=525’C. It is a lighter version of NDN based PUSH support communication scheme especially design for IoT environment. To detect fire disaster, BAM is generated on a fixed seq. no “0” in the PIT. It indicates the occurrence of a fire disaster in a particular SL-1 or SL-2 of a SC as per scenario, discuss in Section 5.

### 4.2. Processing of Consumer and Producer in Normal Mode

In normal mode, when the user (consumer) issues the IM, it is first matched in the CS of the NDN consumer. On a successful match, the producer node responds with the DM else moves to PIT entry check. If a PIT entry is found against the IM, no change occurs in PIT data structure else a new entry is created in the PIT for IM and moves to FIB. The IM is forwarded if it is found in FIB else the IM is broadcast to find next node, shown in right block (NDN Consumer) of Figure 4.

### 4.3. Processing of Consumer and Producer in Disaster Mode

In this Section, we discuss the unique functionality of producer and consumer nodes in disaster mode individually as refer to Section 4.3.1 and Section 4.3.2.

#### 4.3.1. Processing of Producer in Disaster Mode

When the producer (FS) reaches the TL, the BAM (IM) generates on a dedicated seq. no. “0” in our case. Seq. no.“0” is fixed to manage fire disaster situation, it is unusable in a normal NDN-based communication between consumer and producer. Producer broadcasts the BAM (IM) to all consumers that lie within one-hop distance in the network, shown in Algorithm 1. Synthetic interest generator [26] creates a synthetic PIT entry at dedicated Seq. no. “0” location of the PIT on receiving of BAM (IM). If the synthetic PIT entry is successfully created then BAM (DM) is cache in CS for forwarding the unsolicited data on BAM (IM) response. This mechanism is used to alert all nearby consumer nodes else synthetic (IM) is discarded as shown in left block (USER N BAM processing) of Figure 4.

**Algorithm 1** Proposed NDN Module for Producer ρ.**Input:** FS, TL, HC **Output:** BAM sent by the Producer 1:CheckFSthresholdvalue2:CheckHCvalue3:BroadcastStrategy=VIF4:Repeat5:CaseI:FSGreaterthanTLANDHCLessthan16:ρbroadcastBAMtoallnearbyconsumers7:$Consumers\;recieve\;BAM\;at\;Seq.no.\;{}^{\prime }0^{\prime }\;of\;PIT$8:CaseII:FSLessthanTLANDHCLessthan19:NormalNDNCommunication10:Default:11:ConsumerforwardBAMtonextHop12:UntilBAMrecievedatlastnode

#### 4.3.2. Processing of Consumer in Disaster Mode

The consumer nodes receive the BAM (IM) at seq. no.“0” position in its PIT generated by the producer. When the hop count (HC) between consumer nodes in the network is greater than ‘1’, the received BAM (IM) interest packet broadcast to next hop. This process continues until broadcast BAM(IM) to reach all nodes. Along with this, the consumer also maintains its satisfied interest at seq.no “0” in the PIT in disaster case. The temporary PIT entry at seq. no. “0” is removed automatically after the BAM broadcast from the consumer to other nearby NDN consumer nodes, as shown in Algorithm 2. The alert message BAM is broadcast via Wi-Fi protocol IEEE-802.11a. It using multi-hop transmission forwarding scheme that ultimately reaches to all consumer nodes of a SC. After receiving the BAM, mobile consumer nodes move towards the FE-1 depending on its current location, as shown in Figure 3 in order to move towards a secure place.

**Algorithm 2** Proposed NDN Module for Consumer *c***Input:** BAM, HC **Output:** BAM to all Nearby Consumers1:CheckBAMStatus2:CheckHCvalue3:Checkconsumernetworkconnection4:BroadcastStrategy=VIF5:Repeat6:CaseI:bool(BAM)ANDbool(cconnected)ANDHCLessthan17:SatisfiedPITPendingEntry8:BroadcastBAMtoAllnearbyConsumers9:$Remove\;PIT\;Pending\;entry\;at\;Seq.no.{\;}^{\prime }0^{\prime }$10:Default:11:NormalNDNCommunication12:UntilBAMrecieveatlastnode

### 4.4. Performance Metrics

The proposed scheme NDN-DISCA is evaluated on the basis of the following parameters i.e., AD and ATH. In a disaster situation, robust and effective communication between nodes are the major concerns that affect the performance of overall system. In order to calculate the robustness of the system we are using the AD (Time metric) parameter that calculates the overall delay between two nodes (both transmission and propagation) and to calculate the effectiveness of the system, we are using the ATH (Rate Metric) that calculates the overall bit rate inside the data structure of NDN node on the basis of cache hit or cache miss.

#### 4.4.1. Average Delay (AD)

The AD is the ratio of the sum of total delay i.e., propagation and transmission to total time taken of a scenario simulation. It can be calculated through Equatin (1) as follows:

(1) $$AD=\frac{\sum_{1}^{N}\left(Propagation\;Delay+Transmission\;Delay\right)}{Total\;Time\;Taken}$$

where ‘*N*’ represents the total number of consumer nodes.

#### 4.4.2. Average Throughput (ATH)

ATH is the ratio of sum of cache hits in Kbps to the total bits transmitted. It is calculated through Equatin (2) as follows:

(2) $$\begin{array}{c} ATH=\frac{\sum_{1}^{N}\left(hits\times const\right)}{Total\;Bits\;Transmitted} \end{array}$$

where ‘*N*’ represents the total number of consumer nodes and ‘const’ is equal to $\frac{512}{1024}=0.5$ (Multiplying factor to access single location in CS table).

#### 4.4.3. Percentage Throughput Gain (% THG)

%THG is the ratio between THG calculated in Equatin (3) to the ATH of selected scheme denoted with ‘*x*’ with the multiplying constant of 100 in the numerator. It is calculated through Equatin (4) as follows:

(3) $$THG={\left(ATH\right)}_{NDN-DISCA}-{\left(ATH\right)}_{x}$$

(4) $$\begin{array}{c} \%THG=\left(\frac{THG}{{\left(ATH\right)}_{x}}\right)\times 100 \end{array}$$

where ‘*x*’ represent a particular scheme i.e., NDN, un-solicited and long-lived interest

## 5. Simulation Scenarios

**Figure 5:**
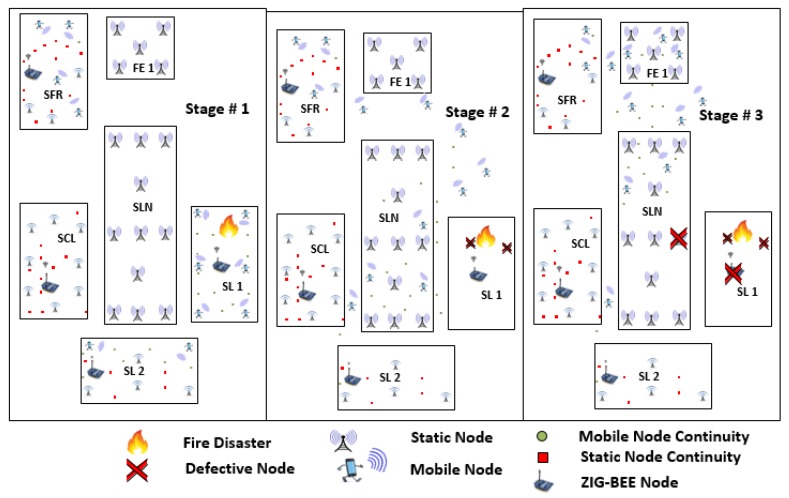
Scenario 1: Smart Lab 1 (SL-1) is Being Affected by Fire Disaster in a Smart Campus.

**Figure 6:**
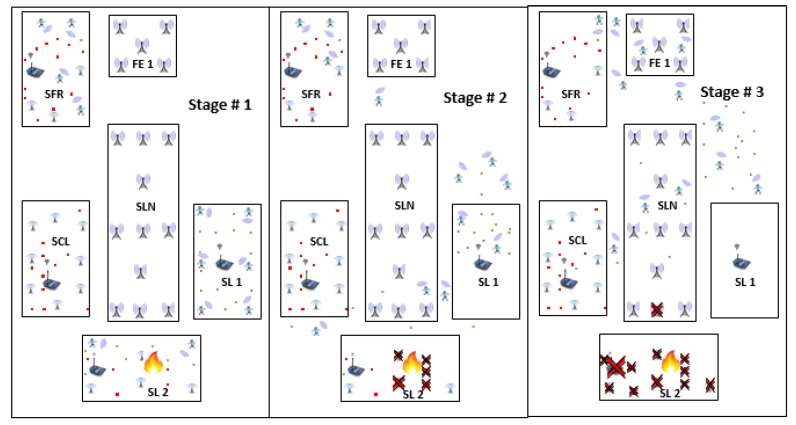
Scenario 2: Smart Lab 2 (SL-2) is Being Affected by Fire Disaster in a Smart Campus.

In this Section, we discuss the two scenarios shown in Section 5.1 and Section 5.2 to show effectiveness and reliability of our NDN-DISCA. It is also used to evaluate the proposed scheme NDN-DISCA under different circumstances such as hybrid NDN node distribution and fire (disaster) affected smart lab cluster as shown in Figure 5 and Figure 6.

### 5.1. Scenario: 1 Disaster Occur in Smart Lab 1 (SL-1)

With reference to Figure 5, it demonstrates the movement of nodes when fire disaster occurs in SL-1 in a SC. Scenario: 1 is shown in three different stages i.e., stage no. 1, stage no. 2 and stage no. 3 based on the movement of the nodes at a regular time intervals. Each stage consists of five blocks represented as SL-1, SL-2, SCL, SFR, SLN, and FE-1. Every block equips with unique features with respect to a node type, nodes distribution, node and block location inside each cluster and the main arena of a SC as mentioned in Table 3. In stage no. 1, fire disaster just occurs in SL-1. In this stage, every node is fixed to its initial rest position, no movement of nodes is shown in this stage. The stage no. 2 shows the in-between state when mobile nodes move towards the FE-1. As mentioned in Section 6, SL-1 only consists of mobile nodes. Due to the mobility of nodes, there is a fewer number of nodes get affected by a fire disaster because these nodes move randomly from their initial position at a constant speed with a regular time interval. In disaster situation and on receiving the BAM, all of these mobile nodes move towards FE-1. In the last stage, almost every mobile node reaches its final destination i.e., FE-1 except few of them that are defective due to disaster.

### 5.2. Scenario: 2 Disaster Occur in Smart Lab 2 (SL-2)

Similarly, with reference to the Figure 6, it demonstrate the hierarchy of nodes when the fire disaster occurs in SL-2 in a SC. Scenario: 2 is also shown in three different stages. According to nodes movement at different time slots. Each stage consists of five block units representing SL-1, SL-2, SCL, SFR, SLN, and FE-1. Every unit equips with its own unique attributes such as node type, nodes distribution, and node location inside each cluster as mentioned in Table 1. In Section 6, SL-2 consist of hybrid nodes, few are static nodes that are stick to its rest positions and few are mobile nodes that move randomly due to the inbuilt mobility support. All of the mobile nodes roams according to the defined network topology. In stage no. 1, the fire disaster just occurs in SL-2. In this stage, every node is fixed to its initial rest position, no movement of nodes is shown in this stage. The stage no. 2 shows the in-between state when mobile nodes move towards the FE-1 while static nodes are still fixed to its initial position. Due to the hybrid nature of nodes in SL-2, there is a fewer number of mobile nodes get affected by fire disaster because these nodes move randomly at a constant speed with a regular time interval. Contrary to this, the static nodes still stick to its initial positions even in a disaster situation. Almost every static node lies in the domain of SL-2 disaster. It is affected due to lack of mobility feature in them. In a disaster situation, on receiving of BAM, all of these mobile nodes move towards FE-1 while static nodes act as BAM forwarder to other consumer nodes. In the last stage, almost every mobile node reaches its final destination i.e., FE-1 and static nodes are defective due to disaster shown in Figure 6.

## 6. Experimental Setup

In the experimental setup, we use an NS-3 simulator-based library named as ndnSIM version 2.4 on Linux operating system (Ubuntu 16.04 (64 bit)) in dual boot mode. Moreover, the machine with specs core i5 with 2.50 GHz processor, along with 8GB RAM, to evaluate the effectiveness of our proposed scheme NDN-DISCA. There are a total of 100 NDN nodes that are scattered randomly in the area of 50 m × 100 m in the form of five different clusters/ blocks. Each cluster consists of 20 NDN nodes located in the area of 20 m × 10 m inside the main arena except SLN that is an open area of 30 m × 10 m as shown in Figure 5 and Figure 6. Each node is equipped with IEEE 802.11a Wi-Fi standard [40]. Clusters represent SL-1, SL-2, SCL, and SFR. All of these clusters are included in the university department collectively represented as SC. Each cluster is configured with some unique attributes i.e., SCL contains only static nodes, SL-1 contains only mobile nodes and SFR and SL-2 consist of hybrid nodes. The mobility of nodes occupied according to RandomDirection2D mobility model. The Nakagami propagation loss model is used to handle multi-path effect. There is only one producer in every cluster that represents fire sensor and remaining all of them act as consumer nodes. IEEE802.15.4 Zig-Bee protocol [40] is installed on all NDN producer nodes. While IEEE802.11a WiFi is installed all NDN consumer nodes.

The proposed scheme NDN-DISCA operates in two modes i.e., normal mode and disaster mode. In normal mode, consumer nodes request data packets from the producer. While in disaster mode, the producer sends a BAM to all consumer nodes when the value of the FS overcomes TL. In case of disaster, critical information is transfer from one cluster to another cluster through multi-hop wireless communication methodology. The proposed methodology adopts Leave Copy Everywhere (LCE) caching approach, along with Least Recently Used (LRU) cache replacement policy. Cache size is maxed set to 1024 locations with 512 bytes for each CS entry to store interest and data packets. Each simulation is run for 200 s. The final result is obtained after averaging of five runs, shown in Table 3. In all simulation scenarios, the TL is set to >=525’C.

## 7. Performance Analysis

In the following text, we represent the performance analysis of our scheme.

### 7.1. Average Delay (AD)

**Figure 7:**
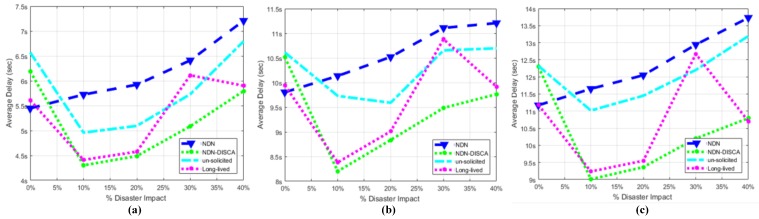
Overall Message (Transmission + Propagation) Delay of SC When Fire Disaster is Being Held in SL-1 w.r.t Three Partition Chunks (**a**) SL-2, (**b**) SCL, (**c**) SFR.

This factor is evaluated with respect to two different scenarios individually. Scenario: 1, only consists of mobile nodes as shown in Figure 5, while scenario: 2 consist of hybrid nodes both static and mobile but in equal proportion shown in Figure 6. The consumer and producer nodes broadcast BAM according to Wi-Fi protocol as mentioned in Section 6. In our proposed scheme NDN-DISCA as shown in Figure 4 and described in Section 4, it is evident that the overall delay factor is reduced. It is due to considering of only one fixed seq no. i.e., ‘0’ in the PIT as compare to native NDN and existing PUSH based schemes (i.e., unsolicited and long-lived) in a disaster environment. Moreover, in native NDN scheme the interest packet is stored in the PIT with respect to multiple seq. no’s. It increases the overall delay due to searching of a particular interest seq no. in the PIT according to a consumer request. Furthermore, existing PUSH schemes also creates an additional payload effect in a disastrous situation, as mentioned in Table 4. Mobility and immobility of nodes also affect on the delay factor. The delay of transferring and receiving the BAM between disaster affected SL-1 nodes to SL-2 and SFR nodes is shown in Figure 7 in three different block segments. Each block segment consists of five independent parameters on *x*-axis i.e., % Disaster impact 0%, 10 %, 20 %, 30 % and 40 %. In Figure 7, block segment 1 represent the transferring and receiving BAM AD curve between SL-1 nodes to SL-2 nodes. Block segment 2 represent the transferring and receiving BAM AD curve between SL-1 nodes to SCL nodes and at last block segment 3 represent the transferring and receiving BAM AD curve between SL-1 nodes to SFR nodes.The Fire disaster doesn’t affect the whole DMS becomes inactive in no time. It depends on the disaster impact factor that destroys the particular number of nodes at a time. The 10 % disaster impact means that only 2 nodes are affected due to fire disaster in overall system. As discussed in Section 6, SL-1 comprised of 20 nodes in total when no disaster occur or in 0% disaster impact and we have total 4 clusters (SL-1, SL-2, SCL, SFR) and an open area SLN.

The total no. of nodes in a whole single scenario is equal to 100 nodes. e.g.,

10% of SL-1 (20) = 2 defected nodes

Remaining available nodes in a whole scenario w.r.t 10% disaster impact = 100 − 2 = 98 nodes Similarly for 20% disaster impact:

Defected Nodes = 20% of Sl-1 (20) = 4

Remaining Available Nodes = 100 − 4 = 96

**Figure 8:**
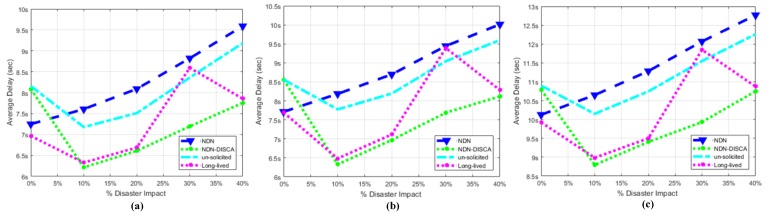
Overall Message (Transmission + Propagation) Delay of SC when Fire Disaster is Being Held in SL-2 w.r.t Three Partition Chunks (**a**) SL-1, (**b**) SCL, (**c**) SFR.

Similar rule is adopted for 30% and 40% disaster impact in both scenarios shown in Figure 7 and Figure 8. Selection of defective nodes in each block segment parameter is chosen randomly through a custom function in ndnSIM. The 0% disaster impact (i.e., normal mode) parameter in each block of Figure 7 acquire more delay than the native NDN. The existence of overhead factor in our NDN-DISCA and existing PUSH schemes mentioned in Table 4 is clearly shown in Figure 7 at 0% disaster impact. This additional overhead delay is due to the continuous monitoring of the FS value in the proposed NDN-DISCA and existing PUSH methodologies. It is not considered in native NDN. After disaster conformation such as at 10% disaster impact parameter, there are instantaneous decline-curves with respect to each scheme are shown in Figure 7 and Figure 8. These are due to the spontaneous effect of our proposed scheme NDN-DISCA and existing PUSH based models in a disaster situation, discussed in Section 4. After that the AD gradually increases with respect to disaster impact parameter. As more and more nodes becomes discarded due to disaster, the more delay we got to reach BAM to every cluster shown in Figure 7 and Figure 8. The additional payload effect due to RTO and vRTO timer effect is clearly visible in each existing PUSH schemes. At 30% disaster impact parameter, there is an instantaneous change in long-lived PUSH scheme in a smooth path as shown in Figure 7 and Figure 8. It is the expiry of vRTO timer effect which needs the re-transmission of packets for calculation of τ parameter in the long-lived interest scheme. It ultimately results in additional AD in overall SC. We used the constant τ parameter in long-lived interest scheme in our scenario. This configuration setup is used for better visualization of vRTO timer effect at 30% disaster impact, shown in Figure 7 and Figure 8 respectively. These sudden delaying effect is not affordable in DMS. As critical information is propagated from one cluster to another cluster, we need a smooth flow of information propagation between nodes for effective response. Moreover, our proposed scheme NDN-DISCA becomes more effective, suitable and robust than legacy NDN and existing PUSH based communication schemes in a disaster environment. Delay is reduced by a margin by implementing our proposed methodology NDN-DISCA. It is also evident in Figure 7 that shows the clear benefit of using the light version of PUSH support in IoT environment for disaster detection i.e., NDN-DISCA. Similarly, in scenario 2, the same nature of results are evaluated after analysis as compared to legacy NDN and existing PUSH schemes as shown in Figure 8 and Equatin (1). The major difference between two scenarios is the delay range on the *y*-axis that is obvious due to the difference in scenario setup as discussed in Section 5. In addition, Figure 7 and Figure 8 also shows that mobile nodes require less time to propagate BAM and become more reliable due to its mobility feature than barely static nodes.

### 7.2. Average Throughput (ATH)

**Figure 9:**
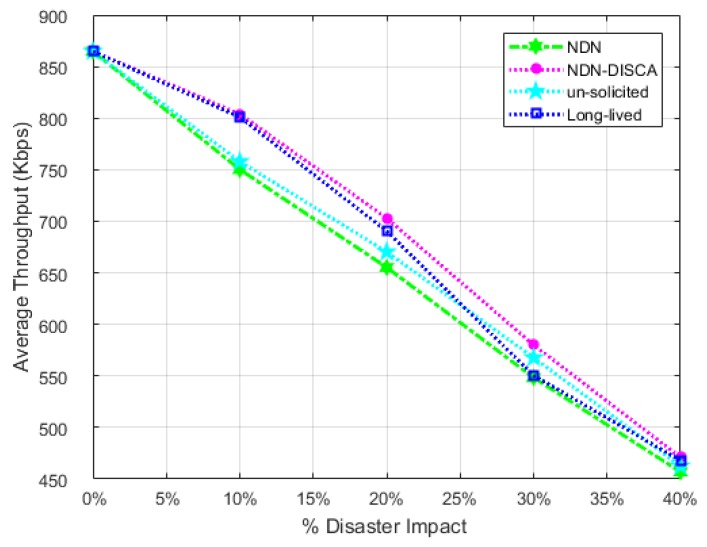
Average Throughput w.r.t Fire Disaster Affected SL-1 in SC.

This parameter is also evaluated with respect to scenario: 1 and scenario: 2 separately in a similar fashion, as stated in the AD. It is calculated in the form of five independent parameters on *x*-axis i.e., % Disaster impact 0%, 10 %, 20 %, 30 % and 40 % with respect to SL-1 and SL-2, as stated above for AD. ATH of scenario: 1 is shown in Figure 9. In scenario: 1, it is analyzed that the throughput is increased minutely in our proposed scheme i.e., NDN-DISCA as compared to NDN and existing PUSH based schemes mentioned in Table 4.

**Figure 10:**
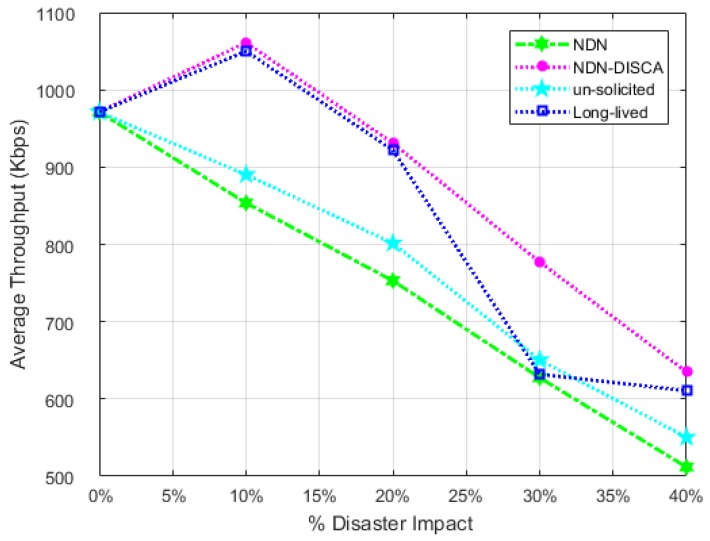
Average Throughput w.r.t Fire Disaster Affected SL-2 in SC.

On 0% disaster impact, the proposed scheme NDN-DISCA, legacy NDN and existing PUSH schemes have the same overall throughput. The unusual behavior at 0% disaster impact represent disaster free scenario at which our proposed scheme NDN-DISCA and comparable PUSH models operates in a normal mode. There is no additional overheard factor included in a throughput calculation for FS status monitoring as in AD. It is due to the fact that FS value monitoring doesn’t affect the throughput factor it only considers in AD of NDN nodes. Once the disaster is detected there are sharp peaks of NDN-DISCA and long-lived schemes prominent at 10% disaster impact in both scenarios. It is more clearly visible in scenario: 2 shown in Figure 10. It is due to the rapid transmission of bulk of critical information in the form of BAM between different clusters mentioned in Section 6 in our proposed scheme NDN-DISCA and existing long-lived interest PUSH model. Moreover at 30% disaster impact, there is a sharp decline in a throughput curve with respect to long-lived PUSH scheme which highlights the vRTO timer effect on SC. Once the vRTO timer resumes, the throughput parameter again increases and goes back on a smooth path. This process continues for N no. of nodes in a SC. Furthermore, with the increase in disaster impact parameter more number of nodes get discarded due to disaster. In response throughput becomes decrease gradually as shown in Figure 9 and Figure 10. Scenario: 1 is less affected by the throughput parameter in a disaster situation as compared to legacy NDN and existing PUSH schemes. It is due to the presence of only mobile nodes in SL-1. As mobile nodes move randomly inside and outside the cluster, it is very difficult to acquire all bits in the NDN nodes every time. Speed mobility is also one of the controlling parameter in mobile nodes, which will address in the future work of this research. Our proposed scheme NDN-DISCA excel legacy NDN scheme and existing PUSH schemes significantly with respect to throughput metric in both scenarios. It can also be proved theoretically in Section 4 and numerically through Equatin (2). It also states that less number of bits become discarded due to unreachable towards CS, PIT, and FIB data structure of nodes as compared to legacy NDN and existing PUSH mechanism. In NDN-DISCA, seq no. `0’ is fixed only for handling disaster situation, so BAM is generated according to seq no. `0’ PIT location only. On the other hand in legacy NDN, interest packet is generated according to consumer demand on various seq. no’s. Further, in existing PUSH schmes RTO and vRTO timer factor minimize the overall throughput in the IoT environment for disaster detection as shown in Figure 9 and Figure 10. The throughput of scenario: 2 also depicted similar nature of results as in scenario: 1. The prominent factor to be noted, there is a wide range of gap between NDN-DISCA ,NDN and PUSH schemes throughput curves. It further clarifies the effect of hybrid nodes distribution in scenario: 2. As static nodes are stick to its initial location, so there is less probability to discard bits while receiving in the NDN nodes. It increases the overall throughput parameter in the proposed DMS NDN-DISCA. It can also be clearly visualize the effectiveness of NDN-DISCA on NDN and existing PUSH schemes as shown in Figure 10.

### 7.3. Percentage Throughput Gain (% THG)

**Figure 11:**
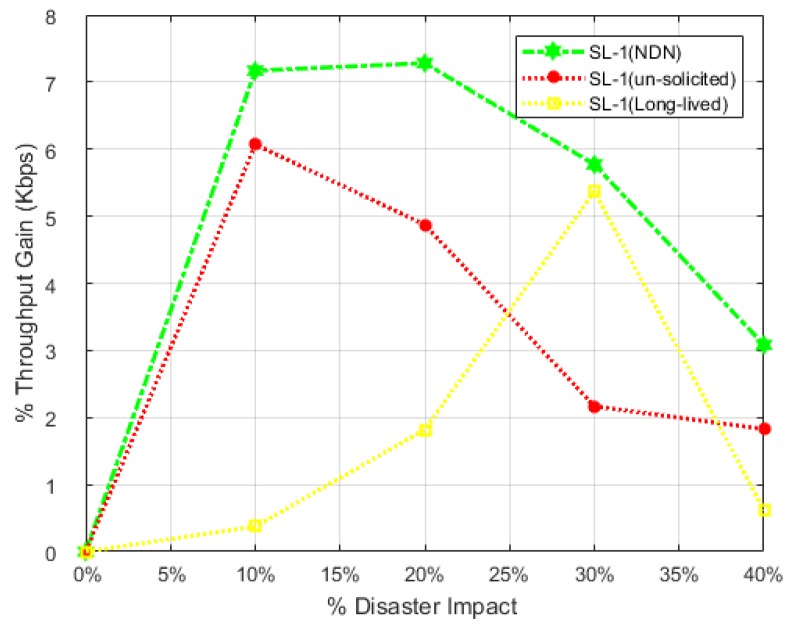
Throughput Gain w.r.t NDN-DISCA to Existing Schemes i.e., NDN, Un-solicited and Long-lived in SL-1.

**Figure 12:**
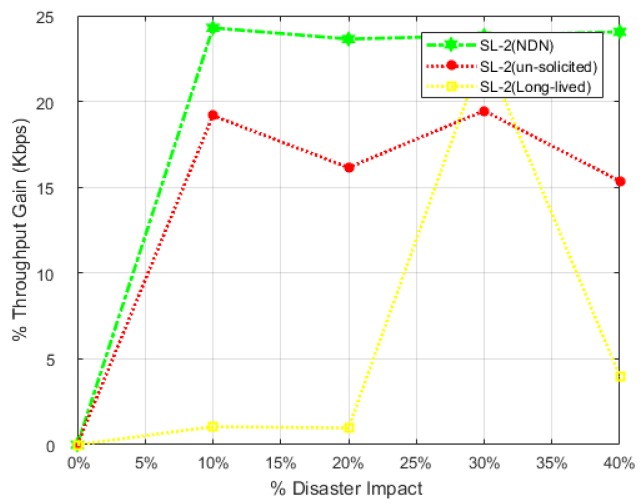
Throughput Gain w.r.t NDN-DISCA to Existing Schemes i.e., NDN, Un-solicited and Long-lived in SL-2.

This factor evaluates the overall gain in throughput with respect to native NDN and existing PUSH schemes in both scenarios: 1 and scenario: 2 individually. The effectiveness of NDN-DISCA in the light of throughput parameter is shown in Figure 9 and Figure 10, along with Section 7.2. Figure 11 shows the throughput gain difference with respect to SL-1, while Figure 12 refers to SL-2. These graphs further clarifies the above discussion regarding the effectiveness of throughput parameter in different scenarios in the case of SC. It also emphasizes how hybrid nodes play their role in overall NDN-DISCA in case of throughput gain in disaster environment. As discussed in Section 7.2, SL-1 is become less affected by %ATH as compared to legacy NDN and available PUSH schemes architecture than SL-2. In a disaster situation, scenario: 1 %THG curves in each scheme is lower than the scenario: 2 %THG curves shown in Figure 11 and Figure 12. These multiple curves shows the clear visualization of gain between proposed NDN-DISCA and existing PUSH schemes along with native NDN in each scenario respectively. In Figure 11 and Figure 12 the RTO and vRTO timer effect becomes more visible in the form of sharp peaks. Specially, at 30% disaster impact in long-lived interest PUSH scheme in both scenarios, it becomes more prompt and visible as discussed in Section 7.2. These results are also proved numerically through Equatin (4) and theoretically in Section 5 and Section 7.2.

## 8. Conclusions and Future Work

In this study, we propose an NDN-DISCA by enabling push support on the fixed seq no. In order to implement the proposed scheme, we have to modify legacy NDN consumer and producer functionality with the addition to FS and TL monitoring modules. This scheme also modifies the existing NDN data structure i.e., PIT, FIB particularly in disaster mode for better efficiency. In the proposed mechanism, the producer creates a BAM message for the awareness of disaster to all nearby consumer nodes to move towards the safe exit in SC. Our proposed scheme NDN-DISCA outperforms legacy NDN and existing PUSH based architectures as mentioned in Table 4, in terms of better interest packet (propagation and transmission) delay and increase overall throughput for disaster mode. Beside this, it can also be implemented in other smart scenarios e.g., smart city, smart home, smart metropolitan and smart hospital which highlights the effectiveness of NDN-DISCA methodology for handling a disaster situation. The efficiency of NDN-DISCA will be further enhanced by considering the parameters such as memory and broadcasting strategy.

## Figures and Tables

**Table 1:** List of Key Terms Used.

Term	Abbreviation	Term	Abbreviation
BAM	Beacon Alert Message	CS	Content Store
CSR	Camper Server	DM	Data Message
DMS	Disaster Management System	FE-1	Fire Exist 1
FE-2	Fire Exist 2	FIB	Forwarding Information Base
FS	Fire Sensor	HC	Hop Count
IM	Interest Message	ICN	Information-Centric Networking
NDN	Named Data Networking	PIT	Pending Interest Table
RTO	Request Time Out	SC	Smart Campus
SCL	Smart Class	SL-1	Smart Lab 1
SL-2	Smart Lab 2	SFR	Smart Faculty Room
SLN	Smart Lawn	TL	Threshold Limit
VIF	Vanilla Interest Forwarding	vRTO	Virtual Request Time Out

**Table 2:** Summary of Related Research Efforts.

Ref.	App. Scenario	DMS Support	Pull Support	Push Support	For IoT	Remarks
[26]	NDN-based Vehicular Network	✗	✓	✓	✗	Only valid for VNDN Not for IoT scenario
[28]	ICN communication for Disaster Scenarios	✓	✓	✗	✗	IoT and NDN Arch. not supported
[33]	CCN-based Disruptive Scenarios	✗	✓	✗	✗	Cached content in Disruptive CCN Arch. IoT Arch. not supported
[34]	NDN-based Emergency Message Delivery	✗	✓	✗	✗	IoT Arch. not supported
[23]	NDN-IoT based PUSH scehmes	✗	✗	✓	✓	DMS not supported
Proposed scheme	NDN-based DMS for SC	✓	✓	✓	✓	Suitable for IoT Arch. with DMS support

**Table 3:** Simulation Parameters.

Parameter	Value/Name
ICN (Communication Stack)	NDN
Propagation Loss Model	Nakagami Propagation LossModel
Propagation Delay Model	Constant Speed Propagation Delay Model
Technology	WIFI_STANDARD_IEEE 802.11a ZIG_BEE_STANDARD_IEEE 802.15.4
Mobility Model	Random_Disc_Position_Allocator Model for static nodes Random_Direction 2D Mobility Model for mobile nodes
Mobility speed	0.2 s
CS Size	1024 total locations
Each CS entry Size	512 bytes
Seq. Range	0–15
Nodes/lab(room	Max 100, 67(static), 33 (mobile)
Number of nodes (Rooms+Labs+Lawn)	4 × 20 = 80 Lawn = 20
Messages frequency/s	8–10/s
Area (m×m)	50 × 100
Caching Policy	LCE
Replacement Policy	LRU
Simulation Time (s)	200 s

**Table 4:** Analytical Comparison b/w Proposed scheme NDN-DISCA and Existing Push Schemes (Un-solicited and Long-lived); RTO: Request Time Out, vRTO: Virtual Request Time Out, τ: Max. exchange info. b/w Consumer and Producer.

Ref.	Scheme Name	Scope	DMS Support	Theme Working	Remarks
[23]	Un-Solicited Data	Local area	✗	It is based on the RTO factor. The packets discard whenever the RTO timer expires. However, the acknowledge packet (i.e., aData) is not received and the discarded packets are re-transmitted from the producer which results in additional payload.	1: In-feasible routing; 2: Only implementable in LAN; 3: Suitable for single hop network.
[23]	Long-Lived-Interest	Local and wide area	✗	It is based on the value of τ parameter, on which the vRTO parameter calculated which enables the stream of data packets through producer upto vRTO timer expires. The packets are discarded after vRTO expire and above mentioned procedure continues.	1: Locked PIT entries up-to vRTO, which creates congestion; 2: Results, increase in delay due to additional payload; 3: Not suitable for NDN-IoT-DMS environment.
Proposed scheme	NDN-DISCA	Local and wide area	✓	It is based on the synthetic interest based BAM on fixed seq. no. “0” without any additional payload in the form of RTO, vRTO and τ.	1: Suitable for IoT Arch; 2: Limited memory utilization due to fixed seq. no. selection; 3: The greater response time due to lack of additional payload factor.
